# Supplementary material for: Fractalkine Improves the Expression of Endometrium Receptivity-Related Genes and Proteins at Desferrioxamine-Induced Iron Deficiency in HEC-1A Cells
Source: Int J Mol Sci. 2023 Apr 27;24(9):7924. doi: 10.3390/ijms24097924 (PMC10177787; doi:10.3390/ijms24097924)
Supplement: Supplementary file 1 [file ijms-24-07924-s001.zip › ijms-2331226-supplementary.pdf]

Article

Fractalkine improves the expression of endometrium receptivity-related genes and proteins at desferrioxamine-induced iron deficiency in HEC-1A cells

Edina Pandur[1,2*], Ramóna Pap[1,2], Gergely Jánosa[1], Adrienn Horváth[1] and Katalin Sipos[1,2]

1 Department of Pharmaceutical Biology, Faculty of Pharmacy, University of Pécs, Pécs, H-7624, Rókus u. 2.
2 National Laboratory on Human Reproduction, University of Pécs, Pécs, H-7624, Ifjúság útja 20
* Correspondence: edina.pandur@aok.pte.hu

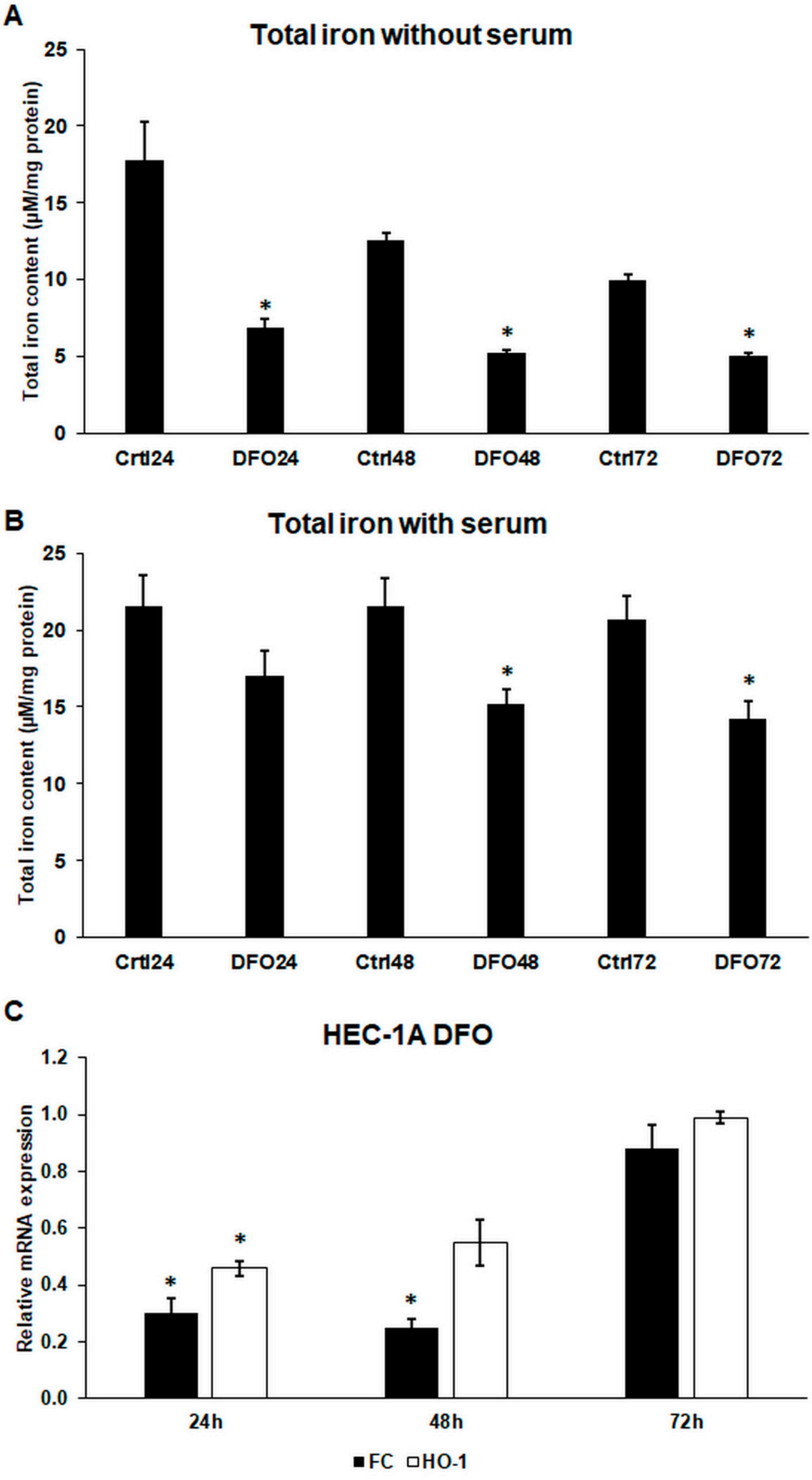
A
Total iron without serum
Total iron content (µM/mg protein)
25
20
15
10
5
0
Crtl24
DFO24
Ctrl48
DFO48
Ctrl72
DFO72
B
Total iron with serum
Total iron content (µM/mg protein)
Crtl24
DFO24
Ctrl48
DFO48
Ctrl72
DFO72
C
HEC-1A DFO
Relative mRNA expression
1.2
1.0
0.8
0.6
0.4
0.2
0.0
24h
48h
72h
FC
HO-1

Supplementary Figure S1. Total iron content and the relative mRNA expression of ferrochelatase (FC) and heme-oxygenase-1 (HO-1) in DFO-treated HEC-1A cells. (**A**) Total iron content of HEC-1A cells treated with DFO in a serum-free culture medium. (**B**) Total iron content of HEC-1A cells treated with DFO in a serum-supplemented culture medium (**C**) Relative mRNA levels of FC and HO-1 of DFO-treated HEC-1A cells. The intracellular iron content was measured by a ferrozine-based spectrophotometric method described by Riemer et al (Riemer et al. 2004). The total iron content was expressed as μM iron/ mg protein. Real-time PCR was performed using an SYBR green protocol. For the normalization of the gene expression levels, GAPDH was used as a housekeeping gene. The untreated cells were used as a control in the experiment. The relative expression levels of FC and HO-1 of the control were regarded as 1. The columns represent the mean ± SD of three independent experiments (n=3). The analysis was carried out in triplicate/sample in each experiment. The asterisk shows $p<0.05$ compared to the control.

Figure 4A

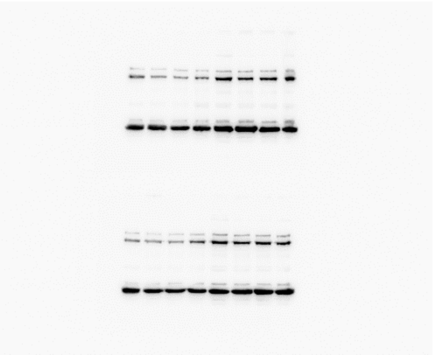

PR and P-PR upper band; GAPDH PR and P-PR lower band

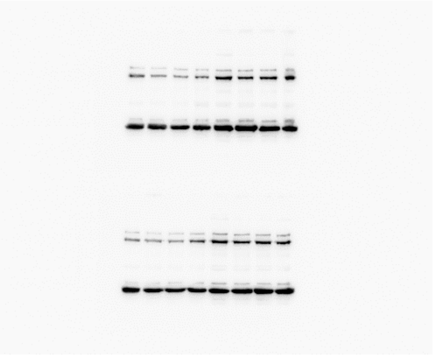

PPR and GAPDH lower blot

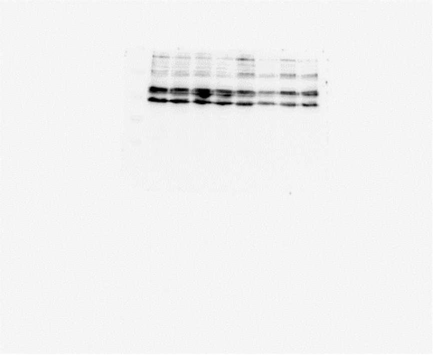

CX3CR1 lower band

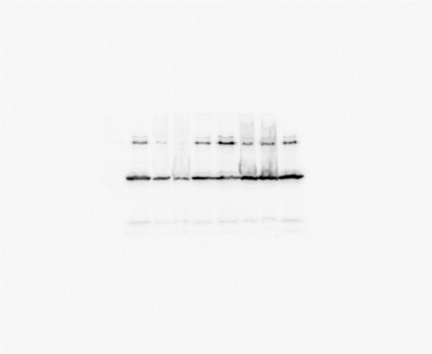

NRF2 upper band and GAPDH

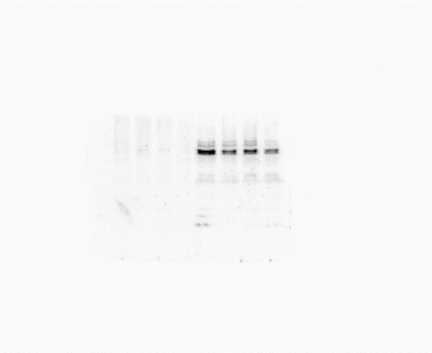

Keap-1

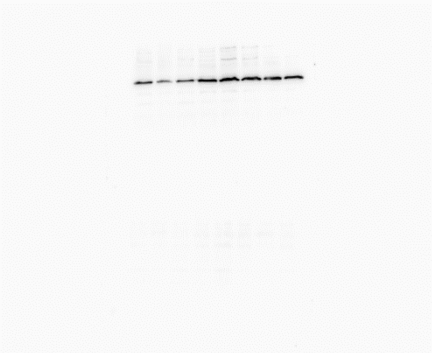

Sox-17

Figure 5A

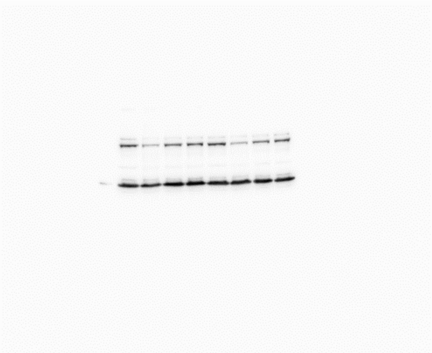

PR and GAPDH

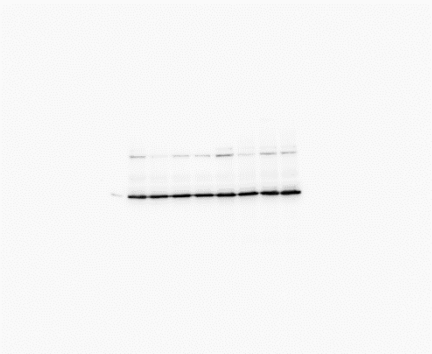

P-PR

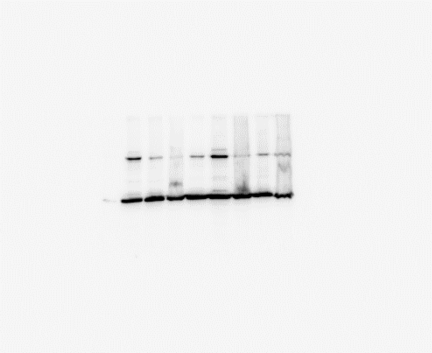

NRF2 upper and GAPDH lower

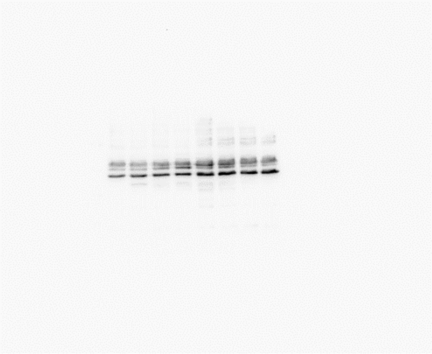

CX3CR1

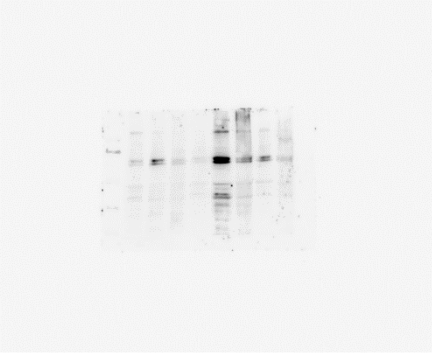

Keap-1

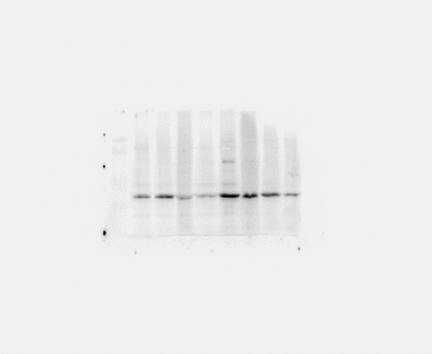

Sox-17

Figure 6A

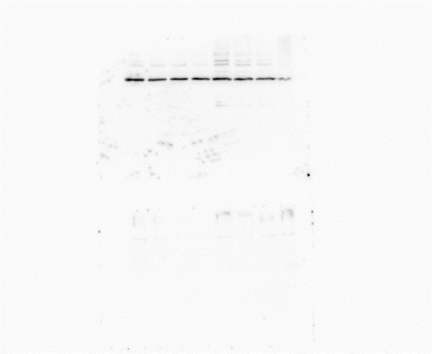

PTGE2R

TIMP2

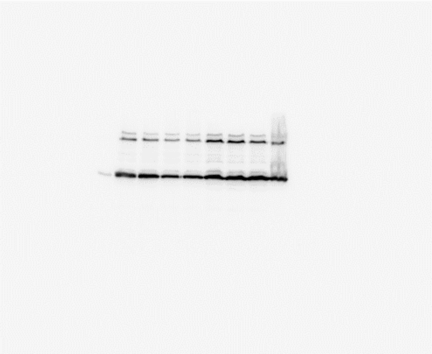

GAPDH

Figure 6B

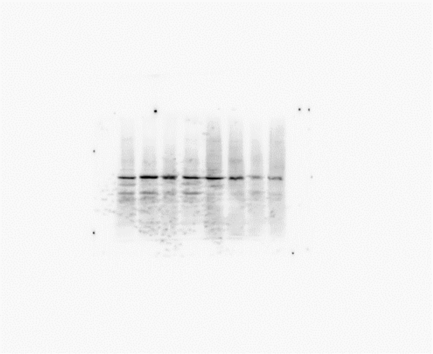

PTGE2R

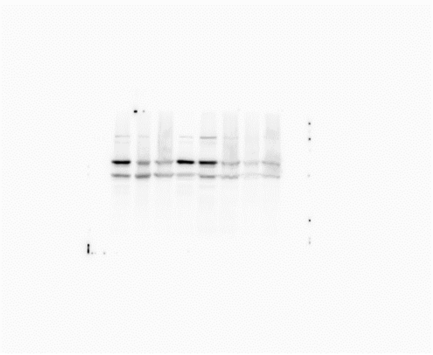

TIMP2

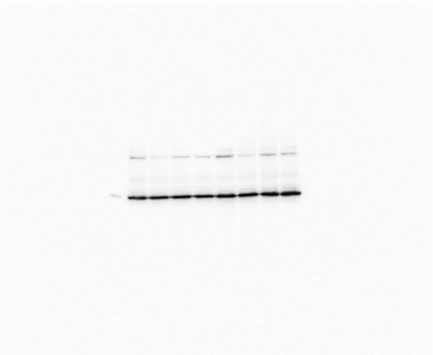

GAPDH

Supplementary Figure S2. Original, full-length Western blot images. The images were taken with a UVItec Alliance Q9 Advanced imaging system using WesternBright ECL chemiluminescent substrate.

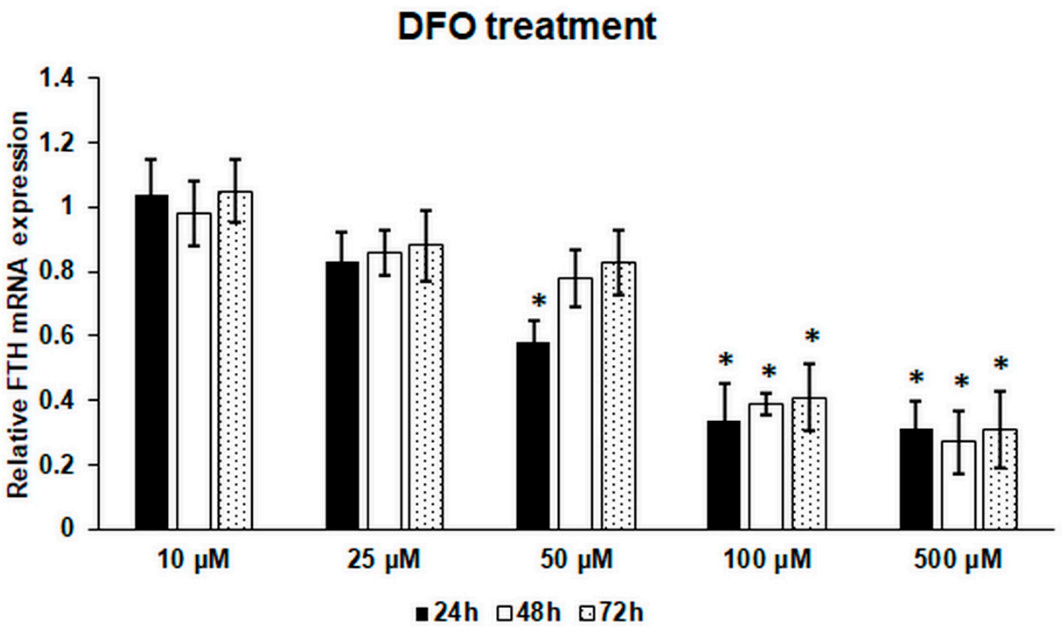

Supplementary Figure S3. Real-time PCR analysis of the ferritin heavy chain (FTH) mRNA expression after DFO treatments. Real-time PCR was performed using an SYBR green protocol. For the normalization of the gene expression levels, GAPDH was used as a housekeeping gene. The untreated cells were used as a control in the experiment. The relative expression level of FTH of the control was regarded as 1. The columns represent the mean ± SD of three independent experiments (n=3). The analysis was carried out in triplicate/sample in each experiment. The asterisk shows $p<0.05$ compared to the control.

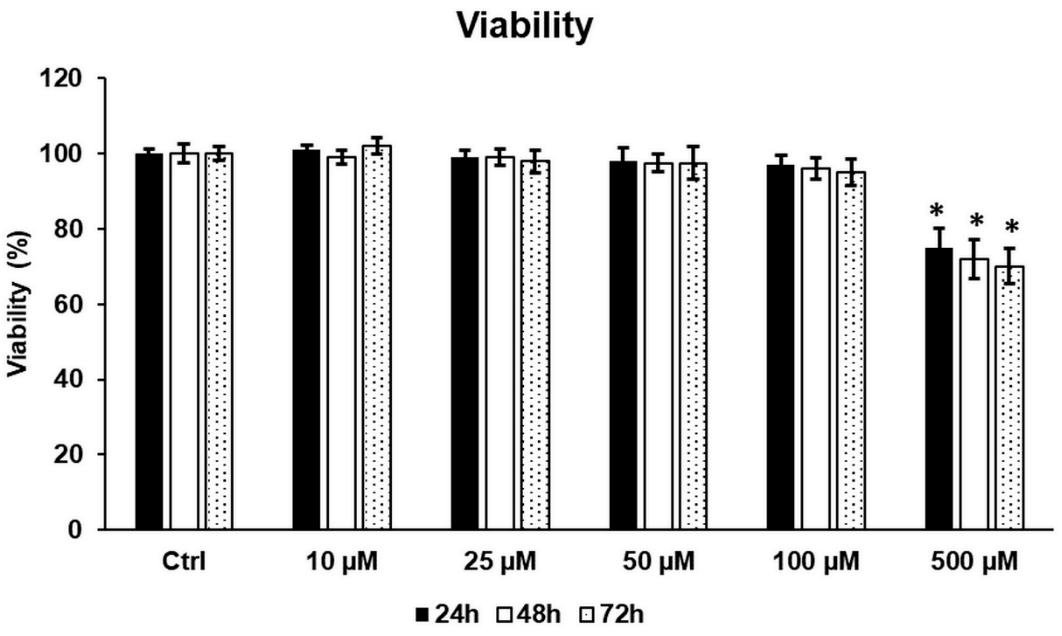

Supplementary Figure S4. Viability measurements of DFO-treated HEC-1A cells. Viability was measured by CCK-8 Cell Viability Assay Kit according to the protocol of the manufacturer. For the examination, $5x10^3$ cells were seeded on a 96-well culture plate. After treatment, 10 µl of WST reagent was added to each well for 1 h. After the incubation, the reaction was stopped by adding 10 µl of 10% SDS to each well. The optical densities were measured by a MultiskanGo spectrophotometer at 450 nm. The columns represent the mean ± SD of three independent experiments (n=3). The analysis was carried out in triplicate/sample in each experiment. The asterisk shows $p<0.05$ compared to the control. The viability was expressed as a percentage of the control.
